# HSV-1 gM and the gK/pUL20 Complex Are Important for the Localization of gD and gH/L to Viral Assembly Sites

**DOI:** 10.3390/v7030915

**Published:** 2015-03-04

**Authors:** Sheung-Yee Kathy Lau, Colin M. Crump

**Affiliations:** Department of Pathology, University of Cambridge, Tennis Court Road, Cambridge CB2 1QP, UK; E-Mail: sykl2@cam.ac.uk

**Keywords:** HSV-1, glycoprotein M, glycoprotein K, UL20, glycoprotein D, glycoprotein H, virus assembly

## Abstract

Herpes simplex virus-1 (HSV-1), like all herpesviruses, is a large complex DNA virus containing up to 16 different viral membrane proteins in its envelope. The assembly of HSV-1 particles occurs by budding/wrapping at intracellular membranes producing infectious virions contained within the lumen of cytoplasmic membrane-bound compartments that are then released by secretion. To ensure incorporation of all viral membrane proteins into the envelope, they need to be localized to the appropriate intracellular membranes either via the endocytic pathway or by direct targeting to assembly sites from the biosynthetic secretory pathway. Many HSV-1 envelope proteins encode targeting motifs that direct their endocytosis and targeting, while others do not, including the essential entry proteins gD and the gH/gL complex, and so it has been unclear how these envelope proteins reach the appropriate assembly compartments. We now show that efficient endocytosis of gD and gH/gL and their incorporation into mature virions relies upon the presence of the HSV-1 envelope proteins gM and the gK/pUL20 complex. Our data demonstrate both redundant and synergistic roles for gM and gK/pUL20 in controlling the targeting of gD and gH/L to the appropriate intracellular virus assembly compartments.

## 1. Introduction

The correct sub-cellular localization of viral envelope proteins is crucial for the assembly of enveloped viruses. This is the case whether viruses acquire their lipid envelope by directly budding through the plasma membrane, or by budding/wrapping at intracellular membranes whereby virions become contained within the lumen of that compartment (with example assembly sites including the endoplasmic reticulum, *trans*-Golgi network, and endosomes). The localization of cellular membrane proteins frequently involves discrete targeting motifs, short sequences present in cytoplasmic domains of such membrane proteins that mediate their inclusion into transport vesicles via direct or indirect interactions with the vesicle forming coat machinery. The best-understood class of cellular targeting motifs is the so-called tyrosine based motifs (YXXΦ, where X indicates any amino acid and Φ indicates an amino acid with a bulky hydrophobic side chain), which interact with clathrin adaptor complexes [1].

Herpesviruses are large and structurally complex DNA viruses that infect a wide variety of vertebrates and cause many clinical and veterinary problems. The envelope of herpesviruses includes many different viral envelope proteins, for example the alphaherpesvirus herpes simplex virus-1 (HSV-1) envelope contains at least 12, and potentially up to 16 different virally encoded transmembrane proteins [2]. HSV-1 envelope proteins mediate a wide range of roles during the virus life cycle and are, thus, important components of the virion. In particular, glycoproteins B (gB), gD and the gH/gL complex play essential roles during virus entry and their incorporation into virions during assembly is vital for the production of infectious progeny [3]. Whilst some herpesvirus envelope proteins such as gB and gE appear to be able to mediate their own localization to intracellular virus assembly sites following *de novo* protein synthesis, others including gD and gH/L appear to rely on other viral proteins for correct localization. Endocytic targeting motifs have been characterized in gB and gE, which allow for trafficking to the cell surface and subsequent internalization where they accumulates intracellularly [4,5]. gD and gH/L however, do not appear to encode any targeting information and expression of gD or gH/L alone gives rise to cell surface localization [6]. This contrasts to the intracellular localization of both gD and gH/L that can be readily observed in infected cells. These observations highlight a requirement for the presence of other viral proteins to localize gD and gH/L correctly.

Previously, it has been demonstrated that gM can efficiently internalize gD and gH/L from the plasma membrane, revealing a mechanism by which gD and gH/L could localize to intracellular virus assembly sites [6]. However, whilst gM can mediate the internalization of gD and gH/gL in transfection assays, subsequent studies suggested that during infection other, gM-independent, mechanisms may also occur. The deletion of the gene encoding gM (U_L_10) from HSV-1 was found to inhibit gH/gL internalization and reduce its incorporation into virions, confirming a role for gM in mediating the appropriate localization of gH/gL to viral assembly compartments. However, no detectable difference in the internalization of gD from the cell surface, or the levels of gD present in purified virions could be observed for the gM-null virus. Also, while gH/gL levels were reduced in gM-null virions, this glycoprotein complex was not completely absent demonstrating at least some gH/gL was still able to reach viral assembly compartments [7]. These data suggest other viral proteins may also be able to localize gD and at least some gH/gL to intracellular sites of HSV-1 assembly.

Previous published data suggest that the gK/pUL20 complex may also be able to alter the localization of other HSV-1 envelope proteins. Cell fusion induced by glycoproteins gB, gD, and gH/gL is inhibited upon co-expression with gK/pUL20, suggesting that this membrane protein complex may be able to alter cell surface expression of these fusion glycoproteins [8,9].

The viral envelope proteins gK and pUL20 are multiple membrane-spanning proteins that are conserved in all alphaherpesviruses. Studies have demonstrated that HSV-1 gK and pUL20 form a complex, and the correct intracellular trafficking, localization and function of these proteins relies on their co-expression [10,11]. The gK/pUL20 complex is important for cytoplasmic virion morphogenesis, as mutant viruses lacking gK or pUL20 accumulate unenveloped capsids within the cytoplasm resulting in a defect in virion egress and spread [12,13,14]. Furthermore, gK and pUL20 are also thought to be important determinants of virus-induced cell fusion, as many different mutations within gK or pUL20 give rise to syncytial variants of HSV-1, which cause extensive cell-cell fusion upon infection [15,16,17,18,19,20]. Structurally, gK has been described to have an *N*-terminal signal sequence and either four transmembrane domains with extracellularly located *N*- and *C*-termini [17,18] or three transmembrane domains with the *C*-terminal tail located intracellularly [21,22]. pUL20 is predicted to lack a signal sequence and to have four transmembrane domains with cytoplasmic *N*- and *C*-termini [23]. gK and pUL20 are both transcribed with late gene kinetics, therefore, requiring viral DNA replication for efficient expression [24,25].

As HSV-1 and pseudorabies virus (PRV) gK/pUL20 have been reported to inhibit cell fusion in gB, gD and gH/gL transfected cells [8,9], this suggests that the cell surface expression levels of at least one of gB, gD, or gH/gL is reduced by gK/pUL20, potentially as a result of internalization. We have now investigated the relative roles of gM and gK/pUL20 for gD and gH/gL localization using transfection-based assays and in cells infected with a series of recombinant viruses lacking gM, gK, and/or pUL20. Our data now show that both gK/pUL20 and gM can mediate gD and gH/L internalization, and that both gM and gK/pUL20 are required for efficient gH/gL incorporation into HSV-1 particles. Interestingly, gD incorporation into virions is mainly dependent on gK/pUL20, despite little effect on gD internalization unless both gM and gK/pUL20 are deleted.

## 2. Experimental Section

### 2.1. Cell Lines and Viruses

COS-7, Vero, VK302 (provided by G Kousoulas, Louisiana State University [13]) and Fd20-1 (provided by G Kousoulas, Louisiana State University [23]) cells were maintained in Dulbecco’s modified Eagle’s medium (DMEM) supplemented with 10% FCS, 2 mM l-glutamine, 100 U/mL penicillin and 100 µg/mL streptomycin. HaCaT cells were maintained in Glasgow Minimum Essential Medium (GMEM) supplemented as above. All viruses lacking gM, gK or pUL20 expression were based on BAC-cloned HSV-1 strain KOS [26] and recombinant viruses were constructed using the primers shown in Table 1 and the two-step Red recombination technique [27]. Viruses lacking gD or gH have been previously described [28,29].

**Table 1:** Primers used for construction of recombinant HSV-1.

COL426	TGCGGTCTGCGTCACGGGGCTCCTCGTCCTGGCCTCTGTGTAATAGTGAGAATTCCCTGCTTTTACGCCACGAGGATGACGACGATAAGTAGGG	Forward primer for replacing codons 53–55 of U_L_10 with three stop codons
COL427	ACCCCGGCATAAGAGCTCGCCGTGGCGTAAAAGCAGGGAATTCTCACTATTACACAGAGGCCAGGACGAGGACAACCAATTAACCAATTCTGATTAG	Reverse primer for replacing codons 53–55 of U_L_10 with three stop codons
COL468	GGTACGCCCCACCGGCACCAACAACGACACCGCCCTCGTGTGATAGTAAGCTTACCAGACCCTATTGTTTCTGAGGATGACGACGATAAGTAGGG	Forward primer for replacing codons 54–56 of U_L_53 with three stop codons
COL469	GGGGGGTGCGTCGGGGCCCCCAGAAACAATAGGGTCTGGTAAGCTTACTATCACACGAGGGCGGTGTCGTTGTCAACCAATTAACCAATTCTGATTAG	Reverse primer for replacing codons 54–56 of U_L_53 with three stop codons
COL464	CCTTCCTCTGGTGGATCGAGATCTGGTCGACGAGGCCGCCTGATAGTAAGCTTAGGGAGAACTGCCACTGGAGAGGATGACGACGATAAGTAGGG	Forward primer for replacing codons 20–23 of U_L_20 with three stop codons
COL465	GAGGACAATGAAAACTGTTCCTCCAGTGGCAGTTCTCCCTAAGCTTACTATCAGGCGGCCTCGTCGACCAGATCAACCAATTAACCAATTCTGATTAG	Revere primer for replacing codons 20–23 of U_L_20 with three stop codons

### 2.2. Antibody Internalization Assays

COS-7 cells grown on glass coverslips were transfected using Trans-IT LT1 according to the manufacturer’s instructions (Mirus Bio, Madison, WI, USA). At 24 h post transfection, cells were incubated with primary antibodies against gD or gH/gL (Table 2) diluted in pre-warmed supplemented DMEM for 1 h at 37 °C in a humidified chamber. Cells were then washed once with PBS before being fixed in 3% (v/v) paraformaldehyde in PBS for 15 min at room temperature and then washed three times with PBS. Cells were permeabilised and blocked using 0.1% TX100/1% FCS in PBS for 15 min. The samples were then incubated with Alexa-647 conjugated anti-mouse secondary antibody (Life Technologies, Grand Island, NY, USA). The coverslips were then washed and mounted onto glass slides using ProLong Gold antifade reagent containing DAPI (Life Technologies). For infected samples, Vero cells grown on glass coverslips were infected with HSV-1 strains at 3 PFU/cell. At 7 h post infection, cells were incubated with primary antibodies against gD or gH/gL for 1 h and fixed and permeabilised as described above. Samples were then incubated with anti-VP5 antibody (Table 2) followed by subtype specific secondary antibodies (anti-IgG2a-Alexa-488 and anti-IgG2b-Alexa-568; (Life Technologies) and mounting on slides. Fluorescence microscopy images were captured using a 60 × 1.4-numerical-aperture oil objective (Olympus, Tokyo, Japan) of an Olympus IX81 inverted wide field microscope using Image-Pro Plus software. Images were processed using ImageJ software (v1.43) and Adobe Photoshop.

**Table 2:** Antibodies used for immunofluorescence (IF) microscopy and Western blotting (WB).

Antigen	Name	Species	Isotype	Application (Dilution)	Source
Actin	AC-40	Mouse	IgG2a	WB (1:5000)	Sigma
gB	CB24	Mouse	IgG2b	WB (1:60)	[30]
gD	LP2	Mouse	IgG2a	IF (1:3)	[31]
	LP14	Mouse	IgG2a	WB (1:10)	[31]
gH	BBH1	Mouse	IgG2a	WB (1:10)	Abcam
	LP11	Mouse	IgG2a	IF (1:3)	[32]
gM	gM-B	Rabbit	-	WB (1:1000)	From: H Browne
pUL20	-	Rabbit	-	WB (1:1000)	From: H Browne
pUL37	-	Rabbit	-	WB (1:5000)	From: T Mettenleiter
VP5	Ab6508	Mouse	IgG2b	IF (1:200); WB (1:1000)	Abcam
VP16	LP1	Mouse	IgG1	WB (1:2000)	Abcam
VP22	AGV30	Rabbit	-	WB (1:2000)	[33]

### 2.3. Single Step Growth Curves

Vero, VK302 or Fd20-1 cells were seeded at 1 × 10^5^ cells/well in 24-well plates. The next day, cells were infected at 10 PFU/cell by incubation for 1 h at 37 °C before inactivating any residual virus by acid wash (40 mM citric acid, 135 mM NaCl, 10 mM KCl, pH 3.0) for 1 min at room temperature, followed by 3 washes with PBS and replacing with fresh supplemented media. At various times post infection, samples were harvested by freezing at −70 °C. Cells were then lysed in their media by three cycles of freeze thawing and total virus titers were determined by plaque assays.

### 2.4. Determination of Plaque Size

For plaque formation analysis, plaque assays fixed and stained with 0.1% toluidine blue solution were scanned at 600 dpi and diameters were determined by Adobe Photoshop in pixels. The average plaque size was determined for 50 plaques and expressed as a percentage of the average KOS WT plaque diameter.

### 2.5. Sucrose Cushion Virus Purification

HaCaT cells growing in 10 cm dishes were infected at 5 PFU/cell by incubation for 1 h at 37 °C before inactivating any residual virus by acid wash for 1 min at room temperature, followed by 3 × PBS washes and replacing with fresh supplemented media. After 24 h, the culture supernatant was harvested and cleared from cellular debris by two consecutive low speed centrifugations (once at 912× *g*, 20 min, 4 °C, Beckman GH-3.8 and then at 17,000× *g*, 30 min, 4 °C, Beckman SW32Ti). 3 mL of the cleared supernatant was then layered on top of 1 mL of 33% (v/v) sucrose cushion. Virus particles were then pelleted by centrifugation (48,600× *g*, 3 h, 4 °C, Beckman TLA-100.3). The pellet was resuspended in 50 µL of SDS-PAGE sample buffer and analysed by Western blotting with various antibodies (Table 2).

### 2.6. Transmission Electron Microscopy (TEM) and Quantification

Monolayers of HaCaT cells in 10 cm dishes were infected at an MOI of 3 PFU/cell by incubation for 1 h at 37 °C and harvested for TEM analysis at 20 hpi. The cells were washed with 0.9% (w/v) sodium chloride and fixed in 2% glutaraldehyde/2% formaldehyde in 0.5 M sodium cacodylate buffer (pH 7.4) for 4 h at 4 °C. The cells were then harvested from the culture dish by scraping into fixative, pelleted and washed in buffer. The cell pellets were treated with 1% osmium ferricyanide for 2 h, before being washed in distilled water and treated with 2% uranyl acetate in 0.05 M maleate buffer (pH 5.5) for 2 h. The samples were rinsed again in distilled water before being dehydrated in graded ethanol, treated with dry acetonitrile and infiltrated with Quetol epoxy resin. Ultrathin sections were examined with a FEI Technai G2 Transmission electron microscope operated at 120 Kv using an AMT XR60B digital camera running Deben software. Samples were processed by the Cambridge Advanced Imaging Centre, University of Cambridge. For TEM quantification extracellular virions, cytoplasmic non-enveloped capsids, cytoplasmic enveloped virions and nuclear capsids were counted in 8 separate cells for each condition.

## 3. Results

### 3.1. gK/pUL20 Cause the Internalization of gD and gH/gL in Transfected Cells

**Figure 1:**
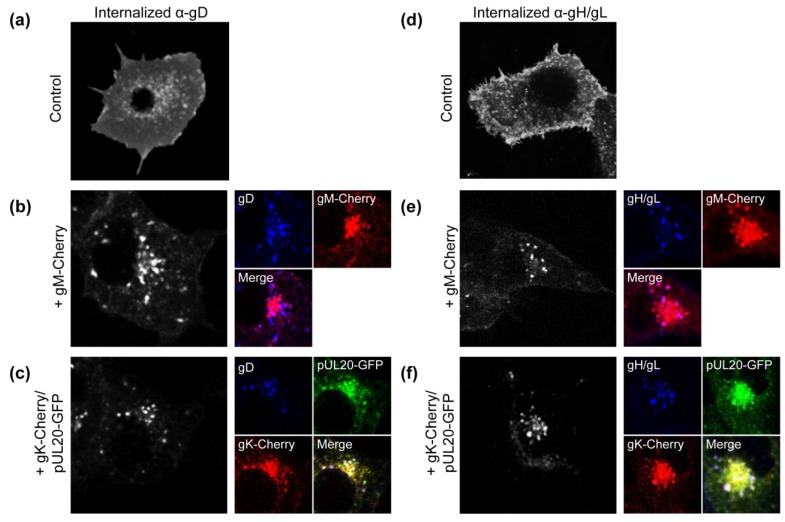
Effect of gK/pUL20 on the internalization of gD and gH/gL. COS-7 cells were transfected with plasmids expressing gD (**a**–**c**) or gH/gL (**d**–**f**); together with plasmids expressing GFP plus Cherry (**a,d**); gM-Cherry plus GFP (**b**,**e**); or gK-Cherry plus pUL20-GFP (**c,f**). At 24 h post-transfection, cells were incubated with gD or gH/gL specific antibodies for 1 h at 37 °C before being fixed, permeabilised and stained with secondary antibody. For clarity, gD and gH/gL signals are shown in black/white in the first column, followed by smaller images of gD and gH/gL signals shown in blue in the second column along with the co-expressed proteins as indicated.

Whilst it is known that gM can mediate the internalization of gD and gH/gL [6], subsequent studies suggest that during infection other mechanisms may also be in place to mediate gD and gH/gL localization to viral assembly compartments [7]. A potential candidate that may be involved in gM-independent mechanisms of glycoprotein localization control is the viral envelope protein complex gK/pUL20. Cell fusion induced by gB, gD and gH/gL is inhibited upon co-expression with gK/pUL20 and one possible explanation for the phenotype is that gK/pUL20 may alter cell surface expression of one or more of these fusion glycoproteins [8,9]. To test this hypothesis, gD and gH/gL were co-expressed with gK/pUL20 or gM and antibody internalization assays were performed. COS-7 cells were co-transfected with plasmids expressing gD or gH/gL in combination with gM-Cherry or gK-Cherry and pUL20-GFP and incubated with gD- or gH/gL-specific antibodies for 1 h at 37 °C before fixation. In this assay, cells are only exposed to the gD and gH/gL-specific antibodies while they are living and intact. Therefore, the only mechanism by which antibody positive signals can occur in intracellular compartments is if gD or gH/gL are internalized from the cell surface after binding to antibody. Control cells expressing just gD or gH/gL demonstrated predominantly cell surface staining, suggesting little internalization of these glycoproteins occurs when they are expressed in the absence of other viral proteins (Figure 1a,d). As expected from previous work, when gM was co-expressed, gD and gH/gL were efficiently internalized from the cell surface (Figure 1b,e). Furthermore, internalised gD and gH/gL showed some co-localization with gM in the perinuclear region (Figure 1b,e, coloured panels). Upon co-expression with gK/pUL20, both gD and gH/gL were also efficiently internalized to a juxtanuclear compartment with little remaining cell surface localization observed (Figure 1c,f). Furthermore, internalized gD and gH/gL showed some co-localization with gK/pUL20 (Figure 1c,f, coloured panels). These results demonstrate that gK/pUL20 and gM are both able to mediate the internalization of gD and gH/gL from the cell surface in transfected cells, and so could serve as alternative and potentially redundant mechanisms for controlling HSV-1 envelope protein localization. However, it is important to note that this assay only monitors copies of gD and gH/gL that pass over the cell surface during the 1 h antibody incubation period, and any gD or gH/gL molecules that remain in intracellular compartments during this time will not be detected. Therefore, while our data demonstrates a clear induction of gD and gH/gL internalization by gM and gK/pUL20, we cannot rule out that gM and gK/pUL20 could also cause some retention of gD and gH/gL in intracellular compartments.

### 3.2. Construction of Recombinant Viruses Lacking gK/pUL20 and gM

**Figure 2:**
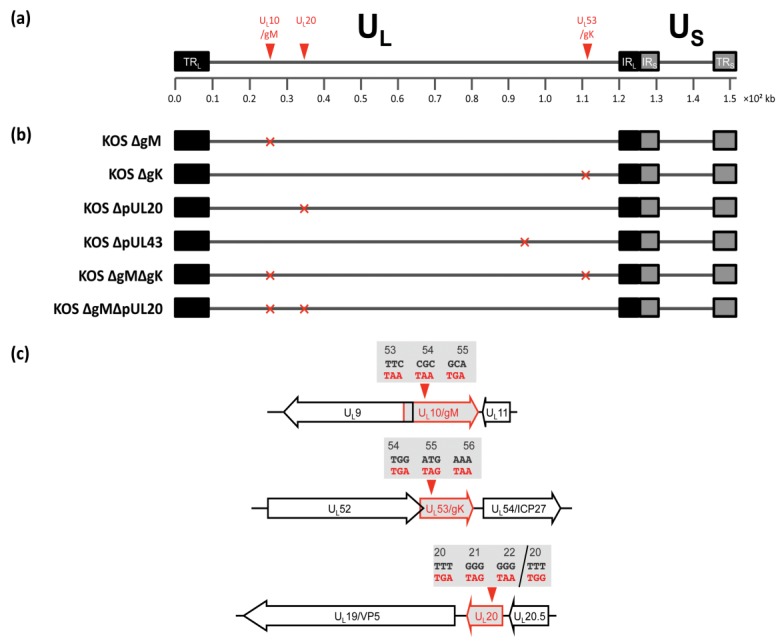
Schematic representation of recombinant viruses constructed. (**a**) Representation of the prototypic arrangement of the HSV-1 genome with the unique long (U_L_) and unique short (U_S_) regions flanked by the terminal repeat (TR) and internal repeat (IR) regions; (**b**) Relative genomic positions of genes mutated to create recombinant viruses; (**c**) Schematic representation of mutations created. Viruses lacking gM, gK, and pUL20 were constructed by replacing codons 53–55, 54–56, and 20–22 with three stop codons in the U_L_10, U_L_53, and U_L_20 genes, respectively.

To investigate the role of gK/pUL20 in gD and gH/gL localisation in the context of infection, single deletion viruses lacking the expression of functional gK or pUL20 were created by inserting three tandem in-frame stop codons into the genes U_L_53 (after codon 53), and U_L_20 (after codon 19) respectively (KOS ΔgK and ΔpUL20; Figure 2). Furthermore in order to investigate possible functional redundancy of gK/pUL20 with gM; a single deletion virus lacking the expression of functional gM was created by inserting three tandem in-frame stop codons into the U_L_10 gene (after codon 52), as well as double deletion viruses lacking the expression of functional gM together with gK or pUL20 were created in the same genetic background (KOS ΔgM, ΔgMΔgK, ΔgMΔpUL20; Figure 2). The short *N*-terminal sequences of gM, gK and pUL20 that could be expressed by these deletion viruses would not be expected to retain any activity, and we would not predict these short peptides to affect viral replication or membrane protein localization. All viruses were constructed using a bacterial artificial chromosome (BAC)-cloned HSV-1 genome (strain KOS; [26]) using the two-step Red-mediated recombination method [27]. All recombiant virus BAC-clones were carefully analysed by restriction enzyme digest to ensure correct recombination had occurred and no detectable deletions or insertions were present. All mutations were verified by sequencing PCR fragments covering the respective regions of the genome that were generated from infected cell DNA. All mutations were present as expected with the exception of the singly mutated KOS ΔpUL20, which had also acquired an additional base insertion of a single G in the first stop codon, leading to a F20W mutation as well as a frameshift rather than the designed stop codons (Figure 2c). The resulting translation product from this mutated gene would contain the first 19 amino acids of pUL20, followed by 23 amino acids from the out of frame sequence before a stop codon is reached. Such a drastically different protein sequence would not be expected to retain any normal pUL20 functionality. The ∆pUL20 mutation in the ∆gM∆pUL20 deletion virus contained the three in-frame stop codons as expected. In HSV-1, the 5' end of the gK-encoding gene (U_L_53) overlaps by 45 bp with the 3' end of the essential DNA synthesis gene U_L_52 (helicase-primase subunit). The mutations introduced into the gK-encoding gene were therefore designed downstream of the U_L_52 stop codon so as to not affect its expression. Likewise the 5' end of the gM-encoding gene (U_L_10) overlaps with the 5' end of the essential origin binding protein gene (U_L_9), which is in the opposite orientation to U_L_10, by 55 base pairs, and so the stop codons were introduced into U_L_10 at 100bp 5' to the U_L_9 start codon to avoid affecting U_L_9 expression. The U_L_20 gene does not overlap with any other known genes. Single HSV-1 gM, gK, and pUL20 deletion viruses have previously been described and constructed by different methods and in different HSV-1 strains [7,13,19,34,35,36,37,38,39]. Whilst the deletion of gM has been reported to have little effect on virus growth, deleting gK or pUL20 results in severe attenuation by approximately 400- and 100-fold respectively, compared to WT virus [36,37]. Therefore, viruses lacking gK or pUL20 require complementing cell lines for propagation of virus stocks (Table 3). The VK302 cell line that expresses HSV-1 gK [13] and the Fd20-1 cell line that expresses HSV-1 pUL20 [23] were used to propagate and titer all ∆gK and ∆pUL20 viruses, respectively. Mutant viruses obtained from these Vero-based complementing cell lines are fully infectious in complementing cells. Infection of non-complementing cell lines can be used to investigate the effects of these gene deletions on post-entry stages of a single round of viral replication.

**Table 3:** Titers of viruses lacking gK or pUL20 in complementing and non-complementing cell lines.

Virus	Virus Titre (PFU/mL)	
	Non-Complementing Cell Line (Vero) *	Complementing Cell Lines (VK302/Fd20-1) **
KOS ΔgK	1.52 × 10^4^	2.63 × 10^6^
KOS ΔpUL20	3.40 × 10^3^	9.70 × 10^5^
KOS ΔgMΔgK	2.20 × 10^3^	1.08 × 10^6^
KOS ΔgMΔpUL20	2.70 × 10^2^	1.53 × 10^5^

Virus stocks, were prepared by infecting complementing cells (VK302 or Fd20-1) at low MOI (0.1–0.01 PFU/cell) for 1 h at 37 °C. After incubation for up to 4 days at 37 °C, the cells were harvested by scraping into the culture medium and centrifugation (4 °C, 1200× *g*, 10 min). The cells were lysed to release cell-associated virus by sonication for 20 s at 39% amplitude in a cup horn sonicator (Fisher Scientific) and virus stocks were stored at −80 °C. Viral titers were determined by plaque assays on Vero cells (*) or on the appropriate complementing cell line (**).

### 3.3. Growth Kinetics of Viruses Lacking gK/pUL20 and gM

**Figure 3:**
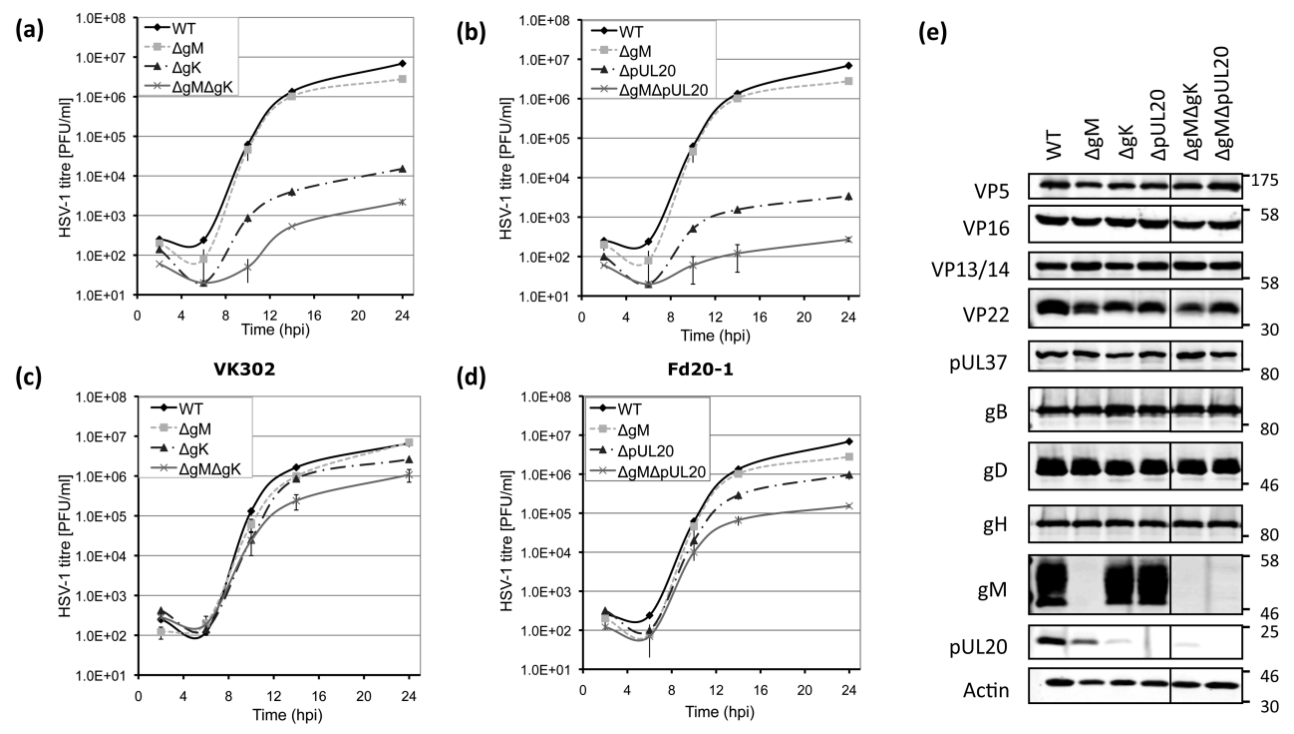

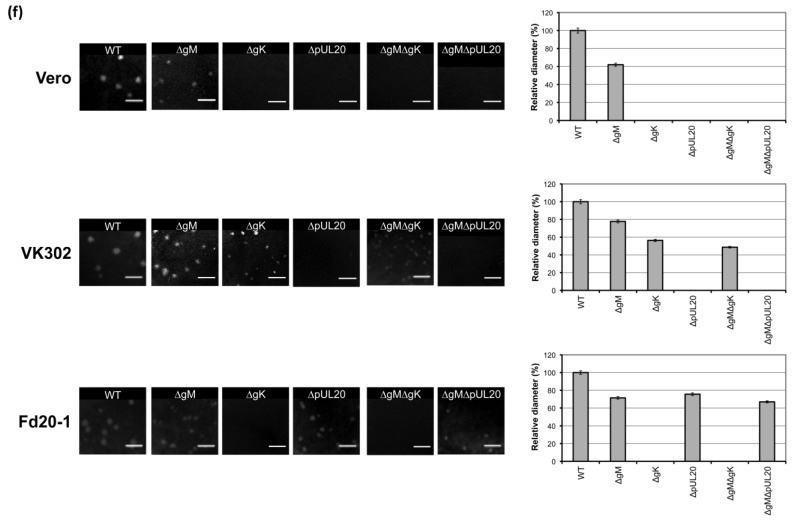
Replication kinetics, plaque formation and protein expression of recombinant viruses. Single-step growth curves at high MOI (10 PFU/cell) were carried out in Vero cells (**a**,**b**); VK302 cells (**c**); or Fd20-1 cells (**d**). Total infectious virus yields were determined by plaque assay using the appropriate complementing cells lines. Error bars represent standard errors of the means of triplicate samples; (**e**) HaCaT cells were infected with the indicated viruses at high MOI (5 PFU/cell) for 24 h before being harvested and lysates separated by SDS-PAGE and analysed by Western blotting using the indicated antibodies. Molecular mass markers (in kDa) are indicated on the right; (**f**) Plaque formation of the indicated viruses was determined by standard plaque assay on Vero, VK302 and Fd20-1 cells at 3 days post infection. Representative images are shown. Scale bars: 2.5 mm. Plaque sizes were determined by scanning assay plates and measurement using Photoshop. The relative diameters compared to WT are shown. Error bars represent standard error (*n* = 50).

To investigate the effect of deleting gM, gK, and pUL20 on virus replication rates, single-step growth curves were performed in Vero cells and the gK- and pUL20-complementing cell lines (VK302 and Fd20-1). In Vero cells, the deletion of gM from the virus resulted in peak titers similar to WT, with a c.a. 2-fold reduction at 24 h post infection (hpi) (Figure 3a). The replication of ΔgK and ΔpUL20 however were severely attenuated, showing approximately 450- and 2000-fold reduction in final titers compared to WT respectively (Figure 3a,b). Furthermore, the deletion of gM with gK or pUL20 resulted in a synergistic defect on replication with a reduction of 3000- and 25,000-fold in final titers respectively compared to WT respectively (Figure 3a,b). The complementing cell lines were able to correct the attenuated phenotypes (Figure 3c,d). However the replication of the deletion viruses was not rescued to WT levels indicating that the complementation provided by the cell lines is not complete, although we cannot formally rule out the possibility that random additional changes may have occurred during the construction of the recombinant viruses that are not fully rescued by these complementing cell lines. In the VK302 and Fd20-1 cell lines, the deletion of gM results in similar final titers compared to WT (Figure 3c,d). These results show that whilst the deletion of gM from the virus has little effect on replication, the deletion of gK or pUL20 results in a highly attenuated virus, as expected from previous publications [7,36,37]. Furthermore, the deletion of gM in combination with gK or pUL20 gives rise to a synergistic defect in HSV-1 replication. The severe attenuation in single-step growth curves observed with both gK- and pUL20-deficient viruses in non-complementing cell lines could be the result of reduced numbers of virions being produced, the production of non-infectious virions, or a combination of the two.

### 3.4. Viral Protein Expression of Recombinant Viruses Lacking gK/pUL20 and gM

To investigate the effect of deleting gM, gK, and pUL20 on viral protein expression, infected cell lysates were analysed by Western blot. This revealed that gM and pUL20 were not expressed in cells infected with the appropriate viruses (Figure 3e). Unfortunately no antibodies that detect gK by Western blotting were available for these studies. However, we noted that deletion of gK resulted in reduction of pUL20 levels, consistent with previously published results that the two form a complex and are reliant on one another for processing [10,11]. The deletion of gM, gK, or pUL20 did not affect overall expression of capsid (VP5) or tegument proteins VP16, VP13/14, and pUL37 in infected cells, although all deletion viruses showed a modest reduction in VP22 expression levels (Figure 3e). Interestingly however, we noted VP22 migrated only as a single band in cells infected with viruses lacking gK or pUL20 in comparison to the doublet observed when infected with WT or ∆gM viruses. This suggests that gK/pUL20 may be involved in post-translational modification of VP22. Furthermore, we also noticed somewhat reduced pUL20 levels in cells infected with ∆gM. Recent published data suggests gM and pUL20 interact [40], and our observations here support a potential interaction and suggest gM is necessary to stabilize pUL20 expression. Similar levels of gB, gD and gH/gL were found in cells infected with all mutant viruses compared to WT (Figure 3e).

### 3.5. Plaque Size Analysis of Recombinant Viruses Lacking gK/pUL20 and gM

The ability of HSV-1 to form plaques reflects its ability to enter cells, replicate and spread to neighbouring uninfected cells. Plaque formation of the mutant viruses was assessed by standard plaque assay on Vero, VK302 and Fd20-1 cell lines. This demonstrated that all mutant viruses exhibited either reduced plaque sizes compared to that of WT or were unable to form plaques (Figure 3f). Plaques formed by KOS ΔgM on all cell lines were approximately 60%–80% of the size of WT. All viruses lacking gK or pUL20 were unable to form plaques on Vero cells, but this was restored in VK302 and Fd20-1 cells, respectively. On the VK302 complementing cell line, plaques formed by ΔgK or ΔgMΔgK showed a reduction of 40%–50% in size compared to that of WT plaques. The comparison of plaque sizes formed by ΔpUL20 or ΔgMΔpUL20 on Fd20-1 cells demonstrated reductions of 30%–40% in size compared to WT. These results indicate that the loss of gM results in a modest deficiency in plaque formation, whilst the loss of gK or pUL20 blocks any detectable spread. The inhibition observed for viruses lacking gK or pUL20 can be restored in complementing cell lines, however similar to that observed with the single-step growth curves, complementation is not complete.

### 3.6. Internalization of gD and gH/gL in Infected Cells

**Figure 4:**
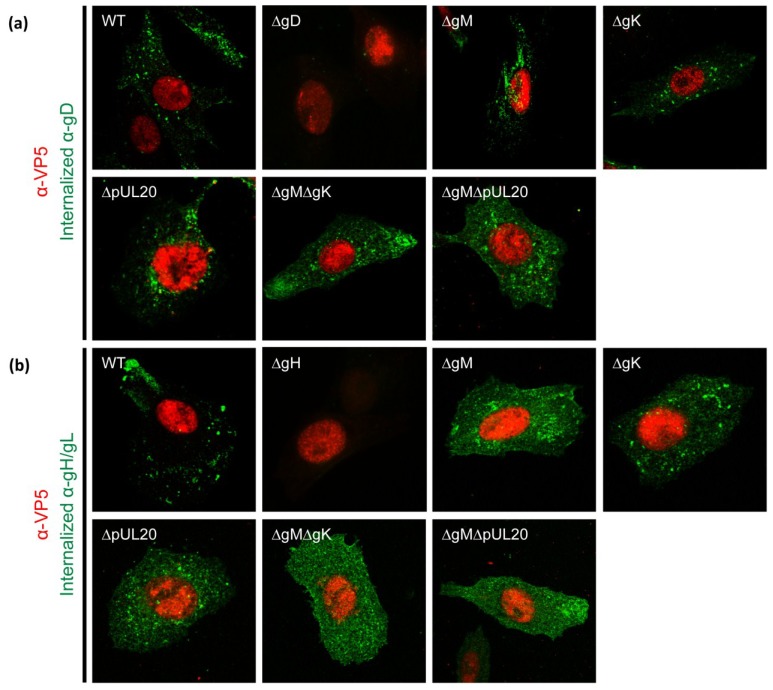
gD and gH/gL internalization in infected cells. Vero cells were infected with viruses as indicated at 3 PFU/cell. At 7 hpi cells were incubated with (**a**) gD or (**b**) gH/gL-specific antibodies for 1 h at 37 °C before being fixed, permeabilised and co-stained with a VP5-specific antibody as a marker for comparable infection.

To investigate the relative roles of gK/pUL20 and gM in the localization of gD and gH/gL in infected cells, antibody uptake assays were performed. At 7 hpi, when virion assembly has just begun, infected Vero cells were incubated with gD or gH/gL-specific antibodies for 1 h at 37 °C before being fixed. As a control for equivalent levels of infection, cells were then permeabilised and incubated with a VP5-specific antibody, followed by subtype specific secondary antibodies. VP5 (the major capsid protein) is a late gene product and localizes primarily to the nucleus. Only cells that demonstrated equivalent levels of VP5 were imaged, and the same exposure settings and post-acquisition processing was used for all samples. No signals were detected for gD or gH/gL antibodies in cells infected with viruses lacking expression of gD or gH/gL respectively, demonstrating the specificity of these antibodies. WT-infected cells demonstrated intracellular staining for gD and gH/L, indicating that both glycoproteins reach the plasma membrane and are internalized efficiently during normal infection (Figure 4a,b). Furthermore, the internalization of gD appeared similar to WT in ΔgM, ΔgK, and ΔpUL20 infected cells suggesting gD can still be internalized in the absence of gK/pUL20 or gM. However there was a clear inhibition of gD internalization for ΔgMΔgK and ΔgMΔpUL20-infected cells (Figure 4a). Additionally, gH/gL internalization was inhibited in all mutant viruses (Figure 4b). These data suggest that different mechanisms are involved in regulating the localization of gD and gH/gL in infected cells. For gD, either gM or the gK/pUL20 complex can independently mediate its internalization, suggesting redundant functions are in place to localize this glycoprotein efficiently. However, the localization of gH/gL appears to require both gM and the gK/pUL20 complex, suggesting gM, gK and pUL20 may function as a complex.

### 3.7. Transmission Electron Microscopy

Consistent with previous publications demonstrating that gK and pUL20 are interdependent on each other for correct localization and function [10], the phenotypes that we have observed upon deleting gK or pUL20 have essentially been the same. Therefore, for subsequent experiments examining the effect of deleting the gK/pUL20 complex from the virus we used HSV-1 lacking gK (with or without gM expression).

**Figure 5:**
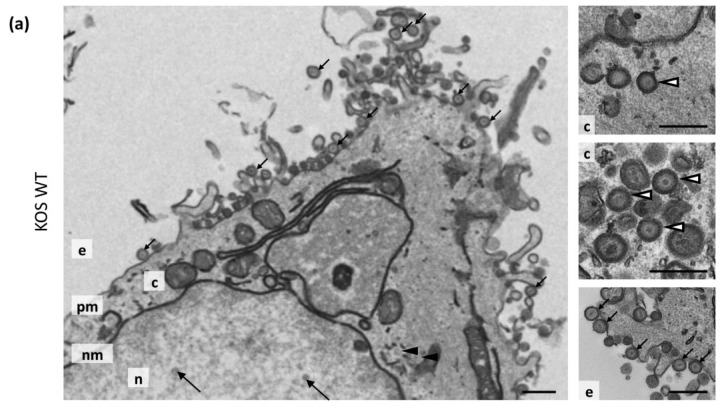

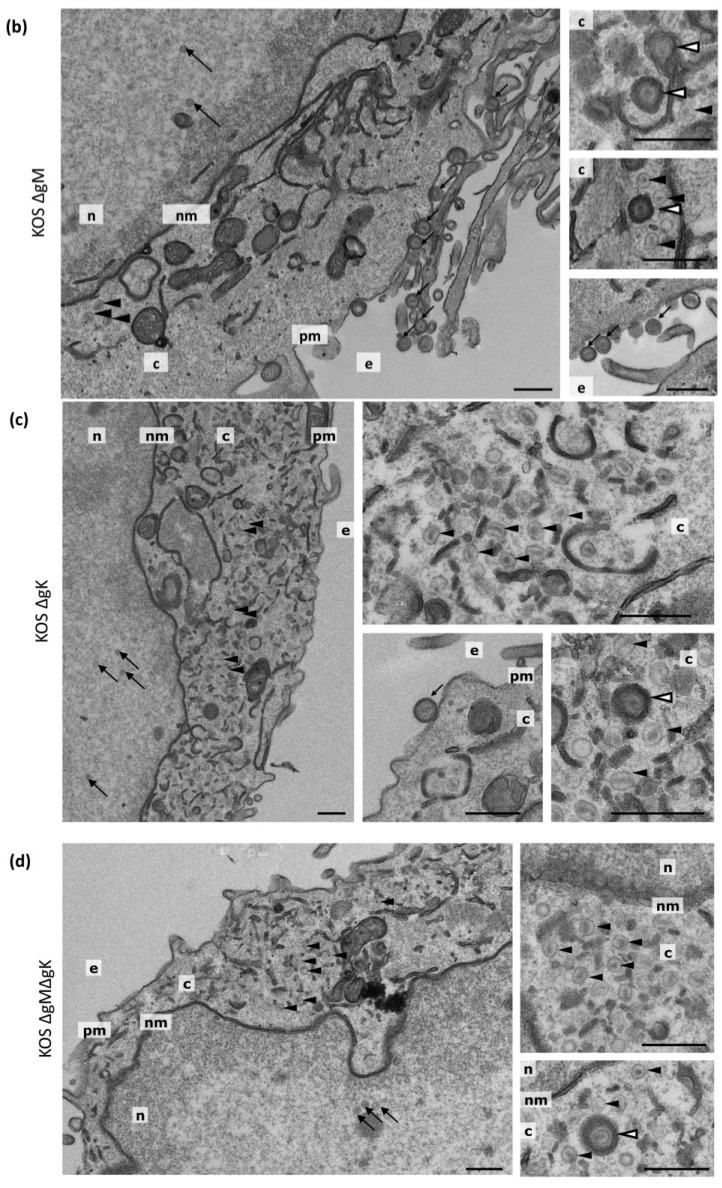
Ultrastructural morphology of cells infected with KOS WT, ∆gM, ∆gK and ∆gM∆gK viruses. HaCaT cells were infected with (**a**) KOS WT; (**b**) ∆gM; (**c**) ∆gK; and (**d**) ∆gM∆gK virus at an MOI of 5 PFU/cell and processed for electron microscopy at 20 hpi. Representative nuclear capsids are marked with large black arrows, extracellular virions with small black arrows, cytoplasmic enveloped virions with white arrowheads and cytoplasmic naked capsids with black arrowheads. The nucleus (n), cytoplasm (c), nuclear membrane (nm) plasma membrane (pm) and extracellular space (e) are marked. Scale bars: 500 nm.

To investigate further the defects of our recombinant viruses, transmission electron microscopy (TEM) analyses were carried out on WT, ΔgM, ΔgK and ΔgMΔgK infected cells. HaCaT cells were infected at an MOI of 5 PFU/cell and processed for TEM analysis at 20 hpi. For each sample, 8 cells were analysed and the numbers of extracellular virions, cytoplasmic non-enveloped capsids, cytoplasmic enveloped virions and nuclear capsids were quantified. The percentages of viral particles relative to the total particles counted were then determined. As expected, cells infected with WT virus displayed a large abundance of extracellular enveloped virions (Figure 5a; Table 4). Furthermore, some cytoplasmic naked capsids were also observed to be present, however these were less common (2.5% of total particles) than cytoplasmic enveloped capsids (9.6% of total particles) indicating the efficiency of normal HSV-1 envelopment (Figure 5a; Table 4). Cells infected with ΔgM were observed to have only small differences compared to WT virus. Overall, there were fewer viral particles in ∆gM-infected cells compared to WT virus, possibly due to delayed entry or replication kinetics of ∆gM as shown before [7]. The ΔgM-infected samples contained a slightly lower proportion of extracellular enveloped virions and slightly higher proportion of cytoplasmic naked capsids (Figure 5b, Table 4), consistent with defects in cytoplasmic envelopment previously observed by others when gM is absent [38]. However, cytoplasmic enveloped virions and extracellular virions were still present indicating that envelopment and egress was occurring relatively efficiently. In contrast, cells infected with the ΔgK and ΔgMΔgK viruses revealed large defects in cytoplasmic envelopment, producing large numbers of unenveloped cytoplasmic capsids and very few extracellular virions or enveloped cytoplasmic virions (Figure 5c,d; Table 4). We noted however that the defect in assembly appeared more severe in cells infected with ΔgMΔgK than the ΔgK virus. These results demonstrate that whilst the deletion of gM from the virus results in a slight defect in virion morphogenesis, the loss of gK/pUL20 function results in a dramatic defect in virus secondary envelopment, although a limited number of enveloped virions are still produced.

**Table 4:** Virus particle quantification from TEM analysis.

Virus	Nuclear Capsids	Cytoplasmic Non-Enveloped Capsids	Cytoplasmic Enveloped Virions	Extracellular Virions	Total
KOS WT	510 (41.9%)	31 (2.5%)	117 (9.6%)	560 (46.0%)	1218
KOS ΔgM	309 (44.5%)	40 (5.8%)	60 (8.6%)	285 (41.4%)	694
KOS ΔgK	244 (47.4%)	258 (50.1%)	9 (1.7%)	4 (0.8%)	515
KOS ΔgMΔgK	186 (34.8%)	347 (64.8%)	2 (0.4%)	0 (0.0%)	535

Virus particles were counted in 8 cells. Numbers in parentheses are the percentage of total particles counted.

### 3.8. Analysis of gD and gH/gL Incorporation into Progeny Virions from Recombinant Viruses

**Figure 6:**
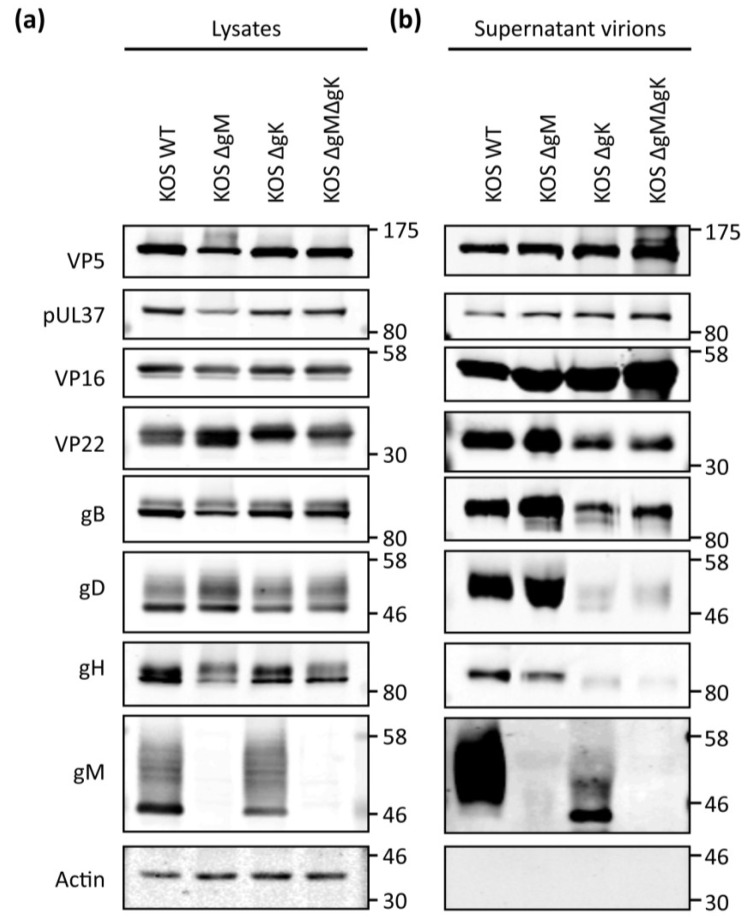

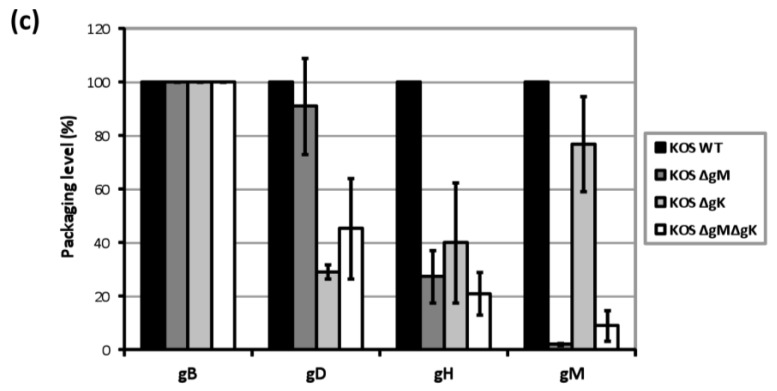
Viral protein incorporation in WT, ΔgM, ΔgK and ΔgMΔgK virions. HaCaT cells were infected at 5 PFU/cell and after 24 hpi, (**a**) cell lysates were prepared and (**b**) progeny viral particles were isolated from clarified cell culture media by centrifugation through a 33% sucrose cushion. Cell lysates and supernatant-derived virions were separated by SDS-PAGE and analysed using the indicated antibodies. Molecular mass markers (in kDa) are indicated on the right; (**c**) Using densitometry, packaging levels were calculated by dividing the amount of protein detected in supernatant-derived virions (normalized for gB) by the amount of protein in supernatant-derived virions of WT virus (normalized for gB). Error bars represent standard error of three independent experiments.

The assembly of infectious progeny virions requires the coordinated recruitment of all envelope proteins to virus assembly sites. Our results suggest that both gM and the gK/pUL20 complex can control gD and gH/gL localization and as a result may function to actively traffic these essential entry glycoproteins to virus assembly sites. In order to investigate the involvement of gM- and gK/pUL20 for the incorporation of gD and gH/L into virions, the viral protein content of progeny virions produced in non-complementing cell lines was examined. The culture media from infected cells was collected, cell debris removed and virus particles partially purified by centrifugation through a sucrose cushion. The relative amount of several viral proteins and cellular actin (as a control for contaminating cellular debris) in the harvested virions, as well as lysates of the producing cells, were analysed by Western blot (Figure 6a,b). Initially, we noted that viral protein levels were dramatically decreased in ∆gK and ∆gM∆gK supernatant virions samples consistent with TEM analysis, which revealed reduced numbers of enveloped virions being produced (Table 4). Loading of supernatant virion samples was therefore normalized to VP5 levels to compare viral proteins present in samples containing equivalent capsid amounts. Actin was not detected in any of the supernatant-derived virion preparations but was easily detectable in cellular lysates, demonstrating a lack of cellular contamination in the isolated supernatant virions (Figure 6b). Relative to VP5 levels, slightly increased levels of pUL37 inner tegument and VP16 outer tegument were observed in supernatant virions for all mutant viruses (Figure 6b). However, VP22 outer tegument and gB envelope levels were greatly reduced in ∆gK and ∆gM∆gK virions (Figure 6b). This suggests fewer fully assembled virions with a complete tegument and envelope are being released from cells infected with these viruses. Interestingly, both gD and gH were barely detectable in ∆gK and ∆gM∆gK virions. The aim of these experiments was to determine the relative incorporation of gD and gH/L into enveloped virions produced by the mutant viruses compared to WT. Therefore given the low level of enveloped virions produced by viruses lacking gK, this causes a potential problem for quantifying the Western blot analysis of the supernatant fraction by normalizing to capsid or tegument protein levels because of contaminating unenveloped and partially tegumented virus particles. In order to control for this, gD and gH levels need to be compared to a protein that is incorporated into the envelope in a gM and gK/pUL20 independent manner. An ideal candidate for such normalization is the envelope protein gB, which has the capacity to localize itself to intracellular compartments irrespective of gM and gK/pUL20 expression. Therefore, the signal levels of glycoproteins present in the isolated supernatant virions were quantified and normalized to gB levels from three independent experiments (Figure 6c). As expected, virion preparations from ∆gM- and ∆gM∆gK-infected cells contained no detectable gM. Little difference was observed in gD packaging of ∆gM virions relative to WT. However a reduction of approximately 72% in gH packaging was observed with ∆gM virions in agreement with our previous work [7]. Virions from ∆gK infected cells contained 70% less gD and 60% less gH than WT. Similarly, ∆gM∆gK virions contained approximately 55% and 79% less gD and gH respectively than WT. Interestingly, the packaging of gM decreased slightly in ∆gK virions, and the gM also migrated faster on protein gels, suggesting possible incorporation of incompletely processed gM in the absence of gK. However, it should be noted that the migration of the highly hydrophobic gM in polyacrylamide gels is somewhat variable. These results indicate that less gD is packaged into progeny virions in the absence of gK but not in the absence of gM, whereas less gH/gL is packaged in the absence of gM or gK. Overall these data suggest that in addition to the clear defect in virus assembly in the absence of gK/pUL20, the virions that are enveloped and released contain substantially lower levels of both gD and gH/L. This reduction in the levels of these essential entry proteins presumably contributes to the dramatic attenuation of HSV-1 in the absence of gK or pUL20 expression. Furthermore, these data suggest the incorporation of gD is more dependent on gK/pUL20, whereas the incorporation of gH/L is dependent on both gM and gK/pUL20.

## 4. Discussion

The aim of this work was to investigate the relative role of gK/pUL20 and gM in the trafficking and localization of gD and gH/gL. Therefore, we generated recombinant viruses lacking gM, gK, or pUL20 singly and in combination in the same genetic background (BAC-cloned KOS strain). To our knowledge no other HSV-1 ΔgMΔgK or ΔgMΔpUL20 deletion viruses have been reported in the literature so far.

Consistent with our previous investigations and other reported gM deletion viruses, we observed that the absence of gM had only minor effects on final infectious virus titers compared to WT (c.a. 2-fold reduction) and also reduced plaque size [7,19,34,38,41]. Previous publications have shown that gK and pUL20 single deletion viruses have a severe attenuation and a major defect in plaque formation. These gK deletion viruses were reported to have reductions in final infectious titres of 400- or 1000-fold [37,39] whereas the pUL20 deletion viruses demonstrated reductions of 100- or 1000-fold [36,39]. The gK and pUL20 single deletion viruses made during this work gave similar reductions in final infectious titres of 450- and 2000-fold respectively. Additionally, consistent with the other studies, we found that our gK and pUL20 deletion viruses were unable to form plaques on non-complementing cell lines.

Consistent with one other study [38], TEM demonstrated that the ΔgM virus produced slightly reduced numbers of extracellular virions and increased numbers of non-enveloped cytoplasmic capsids, suggestive of a role during secondary envelopment. TEM also showed that the deletion of gK caused a profound accumulation of non-enveloped cytoplasmic capsids, demonstrating an important role during secondary envelopment, as previously published by others [39]. However, it is important to note that despite profoundly reduced titers, ∆gK and ∆pUL20 viruses do show some replication, and consistent with a limited level of virus assembly enveloped virions were detected in ∆gK-infected cells by TEM.

Interestingly, we found that the deletion of gM in combination with either gK or pUL20 resulted in a greater defect in virus growth than either single deletion, suggesting potential redundant functions mediated by gM and gK/pUL20. Furthermore, the deletion of gM together with gK/pUL20 resulted in a greater accumulation of cytoplasmic non-enveloped capsids compared to the ΔgM and ΔgK single deletion viruses.

Given the synergistic effect of gM and gK/pUL20 deletion on inhibiting HSV-1 replication, it seems likely that gM and gK/pUL20 may function together in a redundant manner during secondary envelopment. One interpretation of these observed phenotypes is that gM and gK/pUL20 are required for controlling the transport and localization of gD and gH/gL to the virus assembly site. The deletion of gM and gK/pUL20 would, therefore, prevent sufficient amounts of gD and gH/gL being localized to this compartment. As the prevalent model indicates that specific interactions among viral tegument proteins and glycoproteins embedded within membranes are key factors for driving cytoplasmic virion envelopment, a lack of sufficient glycoprotein-tegument protein interactions could prevent secondary envelopment from taking place. Equally, any viruses that do assemble would lack gD and gH/L, thereby rendering them non-infectious. However, there are clear differences in the contribution of gM and gK/pUL20 to assembly; the lack of gM alone does not inhibit secondary envelopment significantly, whereas gK/pUL20 has a more profound effect, suggesting additional roles for gK/pUL20 in mediating virus assembly than redundant functions shared with gM.

The internalization of gH/gL was found to be impaired in cells infected with the single deletion viruses; ΔgM-, ΔgK-, ΔpUL20 as well as ΔgMΔgK and ΔgMΔpUL20-infected cells. This requirement of gH/gL for its internalization suggests that gM and gK/pUL20 may function as a larger complex consisting of all three proteins. Consistent with this, one study has demonstrated that gM interacts with pUL20 and *vice versa* [40]. Interestingly, the internalization of gD was found to differ to that observed with gH/gL; gD internalization was not impaired in cells infected with the single deletion viruses but was upon deletion of gM in combination with gK or pUL20.

One surprising observation during this study was reduced levels of gD packaging in ΔgK virions when there was little/no change in gD internalization in ΔgK- or ΔpUL20-infected cells. This may be explained by a role of gK/pUL20 in additional intracellular trafficking steps once gD has been internalized that mediate its localization to, or retention within, the virus assembly site. Such defects in trafficking steps between intracellular compartments would be difficult to detect by just looking at steady-state levels and internalization from the cell surface, considering the dynamic nature of the endosomal system. In addition, it was interesting to find that the deletion of gK/pUL20 resulted in alterations in the post-translational modifications of VP22. VP22 is one of the most abundant tegument proteins in HSV-1 virions and is a major structural protein. VP22 has been reported to be involved in a number of functions, including interactions with gD and gE/gI during secondary envelopment [42,43] and the reorganisation of microtubules [44]. VP22 has been demonstrated to undergo various post-translational modifications including phosphorylation, glycosylation and ADP-ribosylation [45,46,47]. In infected cell lysates, VP22 can be resolved into at least two migrating forms by SDS-PAGE gel electrophoresis. The slower-migrating form is phosphorylated, whilst the faster-migrating form is non-phosphorylated and is specifically incorporated into purified virions [45,48]. The deletion of gK/pUL20 reported here resulted in a reduction in the amount of faster-migrating VP22 in infected cell extracts. Given the reported interaction of VP22 with gD, this could contribute to the reduced amount of gD packaged into progeny ∆gK virions. However, whilst one study has reported an interaction between endogenous VP22 with gD by co-immunoprecipitation [42], another study was unable to reproduce this under the same conditions tested [49]. To our knowledge, there has been no other finding reporting an involvement of gK/pUL20 in the post-translational modifications of VP22.

Interestingly, ΔgMΔgK-infected cells produce a small number of infectious virus particles, with low but detectable amounts of gD and gH/gL. This indicates a further mechanism by which gD and gH/gL can reach virion assembly sites for incorporation in the absence of gM or gK/pUL20, possibly due to glycoproteins passing *en route* through the virus assembly compartments before they reach the plasma membrane. However, the vast majority of gD and gH/gL clearly requires the presence of gM and gK/pUL20 in order for efficient incorporation.

Several HSV-1 envelope proteins have been shown to internalize from the cell surface in the absence of infection: gB and gE [4,5,50], the gK/pUL20 complex [10] and as shown here, in the presence of gM and gK/pUL20, gD and gH/gL. Due to the dynamics of protein synthesis and trafficking, it seems likely that internalization is required to maintain the steady-state concentration of the viral envelope proteins in the intracellular virus assembly compartments. It is not formally known whether the trafficking of viral envelope proteins over the plasma membrane is a *de facto* requirement for their incorporation into virions, or they can be directly targeted to virus assembly compartments during their transport through the secretory following *de novo* biosynthesis, or both. The ability of a viral envelope protein to internalize can have varying effects on the production of infectious particles, according to the virus and glycoprotein investigated. For example, a single mutation of the VZV gE endocytic motif abolishes virus replication [51], however inhibition of the endocytic machinery, which occurs by 6 hpi during PRV replication, does not affect infectious virus production [52]. Evidence from one study demonstrates that the depletion of Rab5, a GTPase that is involved in trafficking from the plasma membrane to early endosomes, inhibits gD internalization and also significantly inhibits the production of infectious virus particles [53]. The authors conclude that the inhibition of glycoprotein trafficking prevents subsequent virus wrapping, therefore the viral envelope proteins must travel to the cell surface before reaching the final envelopment site. The work presented here demonstrates that in the absence of gM and gK from the virus, approximately 50% and 80% less gD and gH/gL respectively are incorporated into virions. As the absence of gM and gK/pUL20 from HSV-1 also results in impaired gD and gH/gL internalization, this suggests that endocytosis is a major route for targeting these viral envelope proteins to the assembly sites, in agreement with the studies by Hollinshead and colleagues [53]. However, the fact that some gD and gH/gL incorporation still takes place suggests either yet another redundant mechanism in addition to gM and gK/pUL20 allows for gD and gH/gL internalization, or alternatively that some incorporation for gD and gH/L can occur due to proteins reaching the assembly compartment before they reach the plasma membrane.

## 5. Conclusions

In conclusion, our work demonstrates important but differing roles for both HSV-1 gM and the gK/pUL20 complex in mediating the internalization of the viral envelope proteins gD and gH/gL from the plasma membrane, and the targeting of gD and gH/gL to intracellular compartments for incorporation into assembling virions.
